# Surgeon-performed sonographic findings in a traumatic trans-anal rectal perforation

**DOI:** 10.1186/1749-7922-6-26

**Published:** 2011-08-12

**Authors:** Fikri M Abu-Zidan, Mohamed I Abusharia, Katharina Kessler

**Affiliations:** 1 Head Trauma Group, Faculty of Medicine and Health Sciences, UAE University, Al-Ain, UAE; 2 General Surgeon, Al-Ain Hospital, Al-Ain, UAE; 3 Visceral, and Proctology Surgeon, Al-Ain Hospital, Al-Ain, UAE

**Keywords:** Rectal trauma, ultrasound, free intraperitoneal air

## Abstract

Early diagnosis and active management of trans-anal rectal injuries is essential for a favorable outcome. Intraperitoneal free air (IFA) is usually diagnosed by an erect Chest X-ray. Point-of-care ultrasound has been recently used to detect IFA. We report a 45-year-old male who presented to the Emergency Department with lower abdominal peritonitis. Surgeon-performed portable point-of-care ultrasound as an extension of the abdominal examination revealed an inflamed omentum with hypoechoic stranding, thickened non compressible small bowel, and free fluid in the pelvis. A transverse abdominal section of the right upper quadrant showed free intraperitoneal air. Rectal examination revealed a longitudinal rectal tear. Laparotomy has confirmed the sonographic findings. There was a 12 cm intraperitoneal tear of the anterior wall of the rectum which was necrotic. This case clearly demonstrates that portable point-of-care ultrasound gives very useful detailed information even when performed by a non radiologist. Surgeons should be encouraged to use point-of-care ultrasound after appropriate training.

## Introduction

Rectal injuries are uncommon. They are mainly caused by penetrating trauma. Early diagnosis and active management of trans-anal rectal injuries is essential for a favorable outcome [1,2]. Intraperitoneal rectal injuries will cause peritonitis, sepsis and even death if not detected early. Intraperitoneal free air (IFA) is usually diagnosed by an erect Chest X-ray [2]. If the erect chest X-ray was normal, then an abdominal CT scan is recommended. Point-of-care ultrasound has been recently used to detect IFA [3,4]. Hereby, we report an unusual case of trans-anal rectal injury in which point-of-care ultrasound was of a great help for an early diagnosis.

## Case presentation

**Figure 1:**
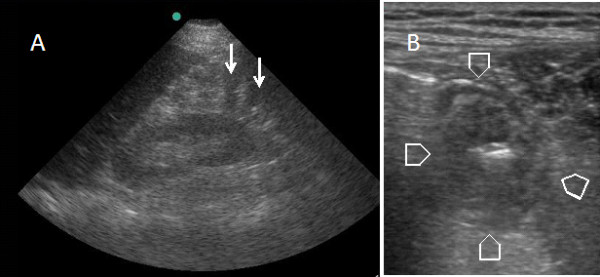
**Surgeon-performed bedside ultrasound showing (A) an edematous omentum with hypoechoic stranding in the right upper quadrant of the abdomen (arrows) and (B) a thickened non compressible small bowel in the right lower quadrant of the abdomen (arrow heads)**.

**Figure 2:**
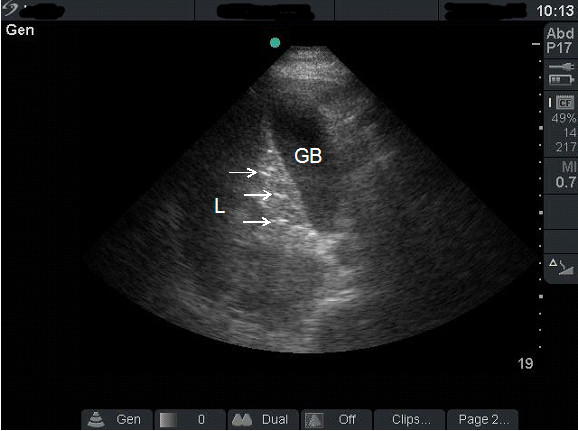
**Transverse sonographic section of the right upper quadrant using a curvilinear probe showing hyperdence echogenic small areas (arrows) between the gall bladder (GB) and the liver (L) indicating free air**.

**Figure 3:**
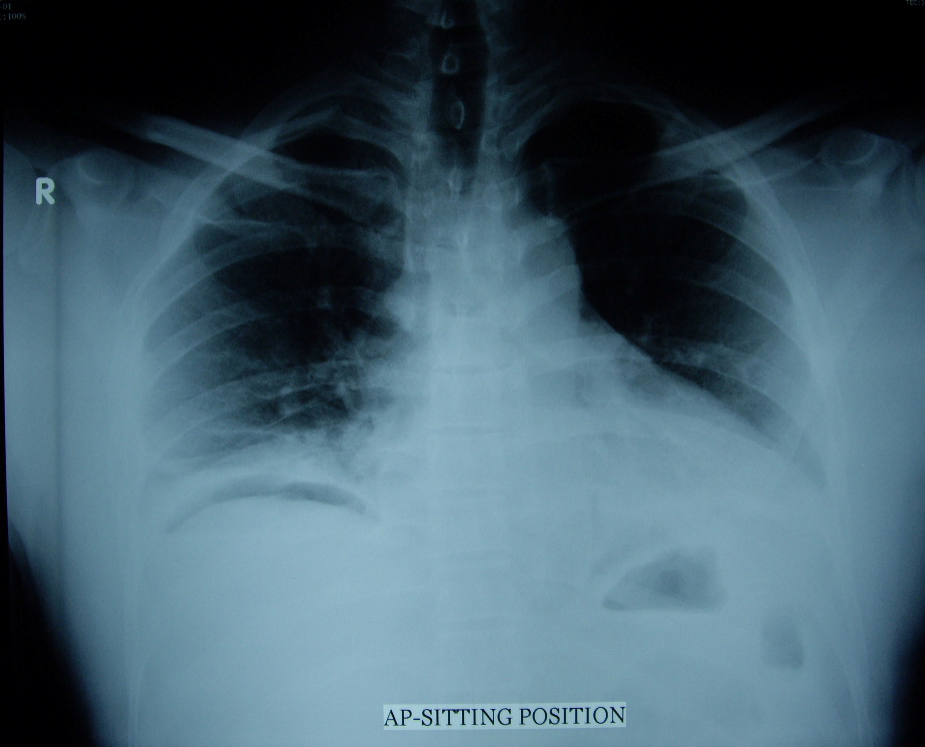
**Erect chest X-ray showing free air under the right diaphragm**.

**Figure 4:**
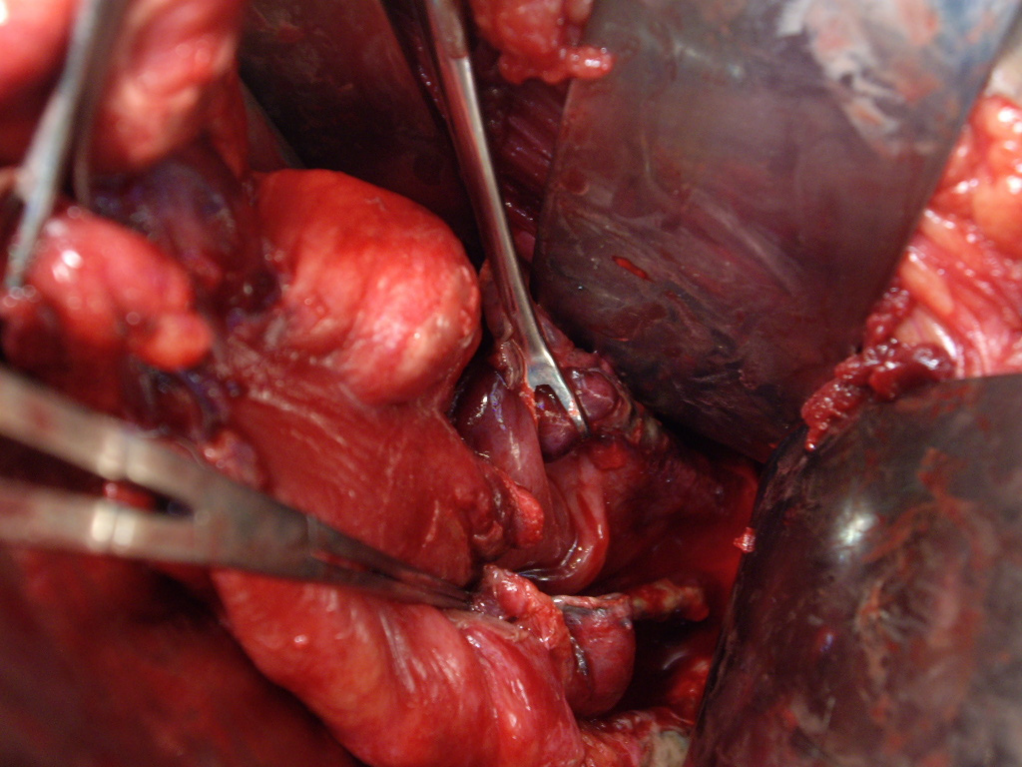
**Laparotomy showing a 12 cm necrotic wound of the anterior wall of the rectum**.

A 45-year-old male presented to the Emergency Department complaining of lower abdominal pain and dysuria of two days duration. His blood pressure was 120/80 mmHg, his pulse was 107 beat per minute and his temperature was 36.8°C. Abdominal examination revealed tenderness and guarding in the lower abdomen. Surgeon-performed portable point-of-care ultrasound as an extension of the abdominal examination was done immediately and revealed an inflamed omentum with hypoechoic stranding in the right upper quadrant (Figure 1A), thickened non compressible small bowel (Figure 1B), and free fluid in the pelvis. A transverse abdominal section of the right upper quadrant showed free intraperitoneal air (Figure 2). Rectal examination revealed a large longitudinal rectal tear 8 cm from the anal verge with an inflamed floppy mucosa. The patient admitted that he has inserted a glass bottle through his anus two days before, which was associated with sudden lower abdominal pain and a small amount of rectal bleeding. Erect chest X-ray confirmed the presence of air under the diaphragm (Figure 3). C-reactive protein was 418 mg/L (Normal less than 0.7 mg/L), serum creatinine was 139 micromol/L (normal less than 107 micromol/l) and white blood cell count was 13.8 × 10^9^/L. Arterial blood gas has shown an arterial oxygen tension of 50 mmHg on normal air. Laparotomy has confirmed the sonographic findings with thickened omentum, an edematous small bowel, pelvic abscess, and a 12 cm intraperitoneal tear of the anterior wall of the rectum which was necrotic (Figure 4). The rectum was dissected and transected 8 cm from the anus. Low mesorectal excision of the necrotic rectum and a Hartman's procedure was performed. Two surgical drains without suction were left in the pelvis. Postoperatively, the patient was ventilated in the ICU. His arterial oxygen tension was 80 mmHg using an oxygen concentration of 50%. The patient received Tazocine intravenously 4.5 gms 8 hourly and Clexane 40 mg subcutaneously daily for one week. His respiratory and renal functions became normal within 4 days. The patient was discharged home on day 10 with good general condition and he is planned for reconnection of the colon after 3 months.

## Discussion

The diagnosis of trans-anal rectal injuries is usually delayed because of patient's denial and late presentation. Some of these injuries are self inflicted or caused by criminal assault [1,2]. High index of suspicion is essential for diagnosis.

In the present patient, portable surgeon-performed point-of-care ultrasound gave very useful information. Point-of-care ultrasound is an extension of the clinical examination. It is a goal-directed study that can be used for rapid diagnosis. It is accurate, non-invasive, cost effective, repeatable, without risk of radiation, and can be done in unstable patients parallel to physical examination and resuscitation [5,6].

It may be argued that ultrasound did not change the clinical management of our present patient. Bedside ultrasound is much quicker when performed by the treating surgeon as an extension of the abdominal examination than doing a formal chest X-ray in the Radiology Department. Furthermore, ultrasound can be done while the patient is in the supine position, and may detect small amount of free intraperitoneal air compared with an erect chest X-ray which may be negative in up to 10% of patients with perforated bowel. Small amount of free intraperitoneal air can be detected under the anterior abdominal wall and in Morison's pouch [7]. This would be useful even in early bowel perforation without peritonitis. Furthermore, ultrasound is useful in disaster and austere situations when formal X-rays cannot be performed [8].

The ultrasound image of IFA results from the reverberation artefact of the ultrasound waves which swings between the ultrasound transducer and the highly reflective air. An increased echogenicity of a peritoneal stripe behind the anterior abdominal wall may be present [3,7,9]. The position of the stripe will change when changing the patient's position. Similar to our patient, trapped free intraperitoneal air bubbles in a localized fluid collection will give rise to echogenic foci [4,7]. The associated findings of thickened omentum and bowel, and free pelvic fluid pointed towards peritonitis in our patient [3,10].

We have performed bedside ultrasound as an extension of the abdominal examination in our patient before performing the rectal examination. Initially the patient denied the history of inserting a foreign body through his anus and he was diagnosed as having lower urinary tract infection in the Emergency Department. He was suspected to have bowel perforation only after the bedside ultrasound was performed.

It is important to stress that ultrasound usually rules in and does not rule out a bowel perforation which indicates that a negative study does not exclude a bowel perforation. FIA detection is operator dependable and can be difficult even for an experienced ultrasound operator [11,12]. The ultrasound findings should be correlated with the clinical picture as a whole and used within defined diagnostic algorithms. If needed, and if the patient was haemodynamically stable, then an abdominal CT scan may give more information than ultrasound [13,14].

It may also be argued that laparotomy would have reached the diagnosis in our patient any way. There are different decisions to be made in cases of peritonitis including the indication for laparotomy and its timing. It would be also useful to collect information about the cause and site of perforation if possible as this may help to decide on what incision to use. Ultrasound may occasionally diagnose the cause of peritonitis, like a perforated duodenal ulcer [4,15].

Early diagnosis and active treatment results in a good prognosis. The good outcome of our patient, despite his multi-organ failure, occurred possibly because of his young age, and active surgical critical care management.

## Consent

Written informed consent was obtained from the patient for publication of his clinical details and accompanying images.

## Competing interests

The authors declare that they have no competing interests.

## Authors' contributions

FA operated on the patient, had the idea, and assured the quality of data collected, drafted the paper, repeatedly edited it, and approved its final version. MA assisted in the operation and follow-up of the patient, helped in the idea, and approved the final version of the manuscript. KK operated on the patient, helped in the idea and drafting of the paper, and approved the final version of the manuscript.
